# Effects of a *N*-Maleimide-derivatized Phosphatidylethanolamine on the Architecture and Properties of Lipid Bilayers †

**DOI:** 10.3390/ijms242316570

**Published:** 2023-11-21

**Authors:** Uxue Ballesteros, Emilio J. González-Ramirez, Igor de la Arada, Jesús Sot, Asier Etxaniz, Félix M. Goñi, Alicia Alonso, Lidia Ruth Montes

**Affiliations:** Department of Biochemistry, Instituto Biofisika (CSIC, UPV/EHU), University of the Basque Country, 48940 Leioa, Spain; uxue.ballesteros@ehu.eus (U.B.); gr.emiliojose@gmail.com (E.J.G.-R.); igor.delaarada@ehu.eus (I.d.l.A.); jesussot@hotmail.com (J.S.); asier.echaniz@ehu.eus (A.E.); alicia.alonso@ehu.eus (A.A.); lidiaruth.montes@ehu.eus (L.R.M.)

**Keywords:** maleimide, membrane fluidity, differential scanning calorimetry, fluorescence, infrared spectroscopy, autophagy proteins

## Abstract

*N*-maleimide-derivatized phospholipids are often used to facilitate protein anchoring to membranes. In autophagy studies, this is applied to the covalent binding of Atg8, an autophagy protein, to a phosphatidylethanolamine (PE) in the nascent autophagosome. However, the question remains on how closely the *N*-maleimide PE derivative (PE-mal) mimicks the native PE in the bilayer. In the present paper, spectroscopic and calorimetric techniques have been applied to vesicles containing either PE or PE-mal (together with other phospholipids) to compare the properties of the native and derivatized forms of PE. According to differential scanning calorimetry, and to infrared spectroscopy, the presence of PE-mal did not perturb the fatty acyl chains in the bilayer. Fluorescence spectroscopy and microscopy showed that PE-mal did not alter the bilayer permeability either. However, fluorescence emission polarization of the Laurdan and DPH probes indicated an increased order, or decreased fluidity, in the bilayers containing PE-mal. In addition, the infrared spectral data from the phospholipid phosphate region revealed a PE-mal-induced conformational change in the polar heads, accompanied by increased hydration. Globally considered, the results suggest that PE-mal would be a reasonable substitute for PE in model membranes containing reconstituted proteins.

## 1. Introduction

Maleimide, or 1H-pyrrole-2,5-dione, is an unsaturated imide that finds numerous applications in organic synthesis and bioconjugation. Maleimides can be bound to polyethylene glycol chains, giving rise to flexible molecular linkers. The maleimide double bond reacts, e.g., with cysteine thiol groups, forming stable carbon–sulfur bonds. In the field of lipids and lipid bilayers, maleimide-functionalized lipids have been developed that allow for polypeptide anchoring to lipid bilayers and membranes [1]. Schuy et al. [2] described the in situ coupling of peptides via a terminal cysteine moiety to maleimide-functionalized phospholipids, thus obtaining specific receptor platforms for functionalized vesicles and nanoparticles.

Autophagy (macroautophagy) is a cytoplasmic process of the degradation of macromolecules and organelles, evolutionarily conserved and of vital importance for cellular and tissue homeostasis [3,4]. This involves the formation of a double membrane around cytoplasmic substrates, resulting in the organelle known as the autophagosome (AP) [5]. Several authors have approached AP expansion using model membrane systems, mostly liposomes of defined lipid compositions and purified proteins [6,7]. Previous work from this laboratory [8,9,10] has applied liposome technology to explore the role of various lipids in AP membrane expansion, in particular in the binding of autophagy proteins belonging to the Atg8 family to lipidic vesicles. The molecular geometry of lipids is important for membrane curvature and thus for bilayer and liposome formation and stability [11]. Lipids with a cylindrical shape, e.g., phosphatidylcholine (PC), give rise to essentially flat lamellae; thus, they are considered as “lamellar lipids” [12]. Conical-shape lipids, e.g., phosphatidylethanolamine (PE), tend to form inverted (water-in-oil) phases, such as the inverted hexagonal H_II_, in excess water [12]. When lamellar and conical lipids coexist in a bilayer, the resulting structure is at the critical edge of bilayer stability; thus, the lamellar structure can be, at least locally in time and space, easily disrupted by a variety of events [8,11]. In the present study, lipid mixtures based on PC and PE have been used, and their stability when PE is partly substituted by a *N*-maleimide-derivatized PE has been explored.

AP expansion requires the conjugation of Atg8 proteins to the polar headgroup of PE at the autophagosomal membrane [13,14]. Landajuela et al. [8] explored this process in a minimal reconstituted system, including a set of recombinant proteins together with synthetic vesicles of defined lipid compositions. Two different approaches, chemical and enzymatic, were used to achieve ATG8-PE conjugation (ATG written in capital letters refers to the mammalian forms of Atg proteins). The chemical process requires inclusion in the liposomal membranes of a maleimide-derivatized PE, namely, 1,2-dioleoyl-sn-glycero-3-phosphatidyl ethanolamine-p-maleimidomethyl-cyclohexane carboxamide (DOPE-mal), to which ATG8 is readily bound. The enzymatically driven lipidation reaction of ATG8 requires the Atg7 and Atg3 proteins, apart from ATP and liposomes. With both methods, a PE-bound ATG8 is obtained. Landajuela et al. [8] observed ATG8-mediated vesicle fusion, which could represent the basis of in vivo AP expansion. Both enzymatically and chemically lipidated forms of ATG8 promoted extensive membrane tethering and fusion. More recently, Ballesteros et al. [10] have examined the binding of LC3C, an Atg8-family protein, to membranes containing PE-mal, with the result that extensive protein lipidation was achieved.

However, the fact that PE-mal and PE have clearly different polar headgroups, which may in turn modify membrane properties, cannot be ignored. In a recent contribution, Maruyama et al. [15] described that enzymatic lipidation of *Saccharomyces cerevisiae* Atg8 on non-spherical giant vesicles induced a noticeable vesicle deformation, although this phenomenon was not observed with chemically lipidated Atg8. In order to clarify any effects of maleimide conjugation on the bilayer properties of PE, we undertook a multi-technique approach in which different membrane physical properties were comparatively studied in bilayers containing either PE or PE-mal, in mixtures with other phospholipids. Our observations indicate noticeable, though not dramatic, differences between the PE- or PE-mal-containing membranes.

## 2. Results

### 2.1. Calorimetric Studies

**Figure 1:**
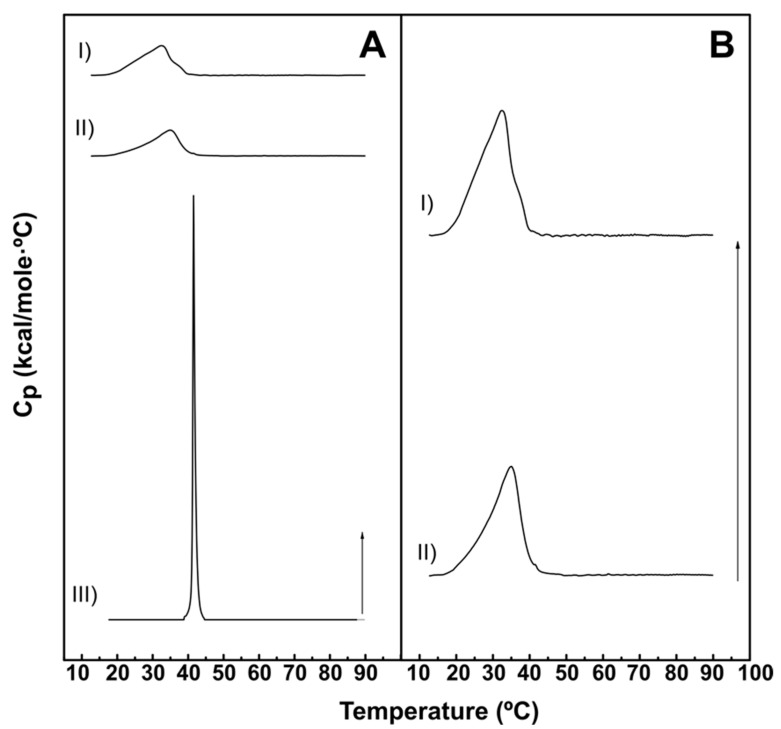
Representative DSC thermograms of lipid:water mixtures based on DPPC. Panel (**A**): DPPC:DOPEmal (70:30) (**I**), DPPC:DOPE (70:30) (**II**) and pure DPPC (**III**). Panel (**B**): DPPC:DOPEmal (70:30) (**I**) and DPPC:DOPE (70:30) (**II**). Thermograms in Panel (**B**) are magnifications from Panel (**A**). Arrows: 2 kcal/mole·°C.

The effect of DOPE-mal when added to lipid bilayers was examined using aqueous dispersions of lipids exhibiting gel–fluid transitions at temperatures close to the physiological range. The thermotropic phase transitions were detected by differential scanning calorimetry (DSC). Thermograms corresponding to pure DPPC and mixtures of DPPC: DOPE-mal (70:30) or DPPC:DOPE (70:30) are shown in Figure 1. Pure DPPC gave rise to the well-known narrow peak at ≈41 °C (Figure 1A(III)), in agreement with previous data [16]. Adding 30 mol% of DOPE or of DOPE-mal caused marked changes in the thermograms (Figure 1A(I,II),B). The transition became much wider, and the transition temperatures were lower. These data are quantitatively shown in Table 1; the transition width is computed as T_1/2_ (band width at half-height) and the transition enthalpy ΔH is obtained from the area under the DSC curve. As seen particularly in Figure 1B, the effects of DOPE and DOPE-mal on the DPPC transition were very similar, although the DPPC:DOPE-mal thermogram looked somewhat less symmetric. Otherwise, both PE lipids caused similar changes in the thermodynamic parameters of the DPPC transition (Table 1).

**Figure 2:**
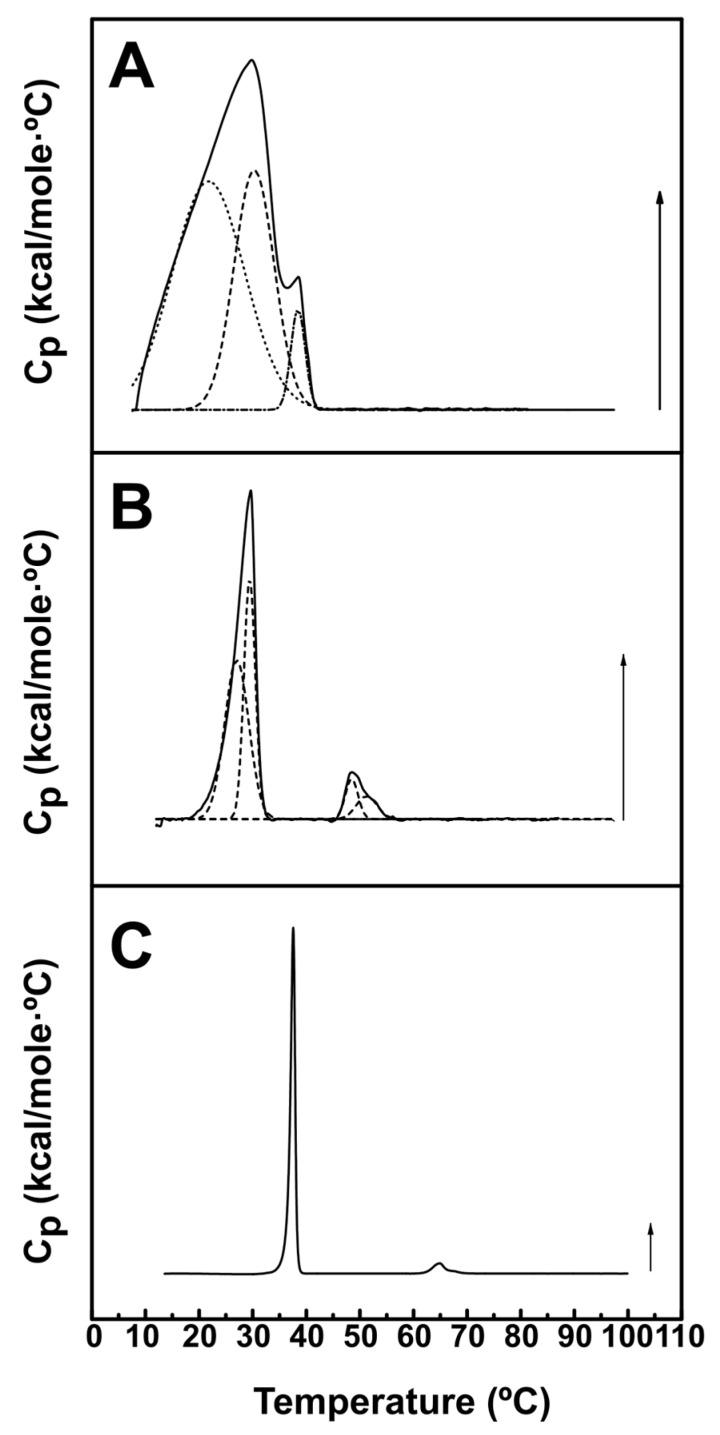
Representative DSC thermograms of lipid:water mixtures based on DEPE. Panel (**A**): DEPE:DOPEmal (70:30). Panel (**B**): DEPE:DOPE (70:30), and Panel (**C**): pure DEPE. Arrows: 0.120 kcal/mol·°C for DEPE:DOPEmal (70:30) and DEPE:DOPE (70:30), and 1.22 kcal/mol·°C for DEPE. The dashed lines indicate the Gaussian components providing the best fit to the experimental overall thermograms.

A further series of DSC experiments was performed with DEPE-based mixtures. Pure DEPE exhibited a gel–fluid transition at ≈38 °C, and a lamellar-to-inverted-hexagonal transition at ≈65 °C (Figure 2C), in agreement with previous studies [17]. The presence of 30 mol% DOPE had the effects of (a) lowering both transition temperatures, (b) widening both transitions and (c) making the thermograms appear asymmetric (Figure 2B). The latter is usually interpreted in terms of poor lipid mixing or of coexisting domains with different lipid compositions [18]. In fact, the thermograms in Figure 2B could be fitted to the sum of two Gaussian lines, whose thermodynamic parameters could be separately computed, and are shown in Table 2. The ΔH associated to each of these components was rather similar, i.e., the areas under the component peaks were of comparable sizes. The effects of 30 mol% DOPE-mal on the DEPE gel–fluid and lamellar–hexagonal transitions were more marked (Figure 2A). In fact, a single, wide and asymmetric peak was observed under these conditions. This complex thermogram could be fitted to three components, whose parameters were also collected in Table 2. The high-T narrow component corresponded probably to the lamellar–hexagonal transition, which PE-mal appeared to facilitate. Moreover, the main gel–fluid transition could be again decomposed into two elements of similar areas (ΔH). Thus, the effect of PE-mal on the stability of bilayers, that appeared to be slight when mixed with DPPC, was more marked with DEPE, a lipid more prone than DPPC to adopt an inverted hexagonal structure.

### 2.2. Infrared Spectroscopy

Infrared (IR) spectroscopy can simultaneously provide information on all the various chemical groups in a molecule, in aqueous media. Particularly relevant in our case are the methylene and phosphate groups of phospholipids. The aqueous dispersions of pure DOPE-mal, ePC:DOPE:PI (35:55:10) and ePC:DOPE:DOPE-mal:PI (35:25:30:10) were examined. These mixtures have been used, in this and other laboratories, in in vitro autophagy studies [6,8,10].

**Figure 3:**
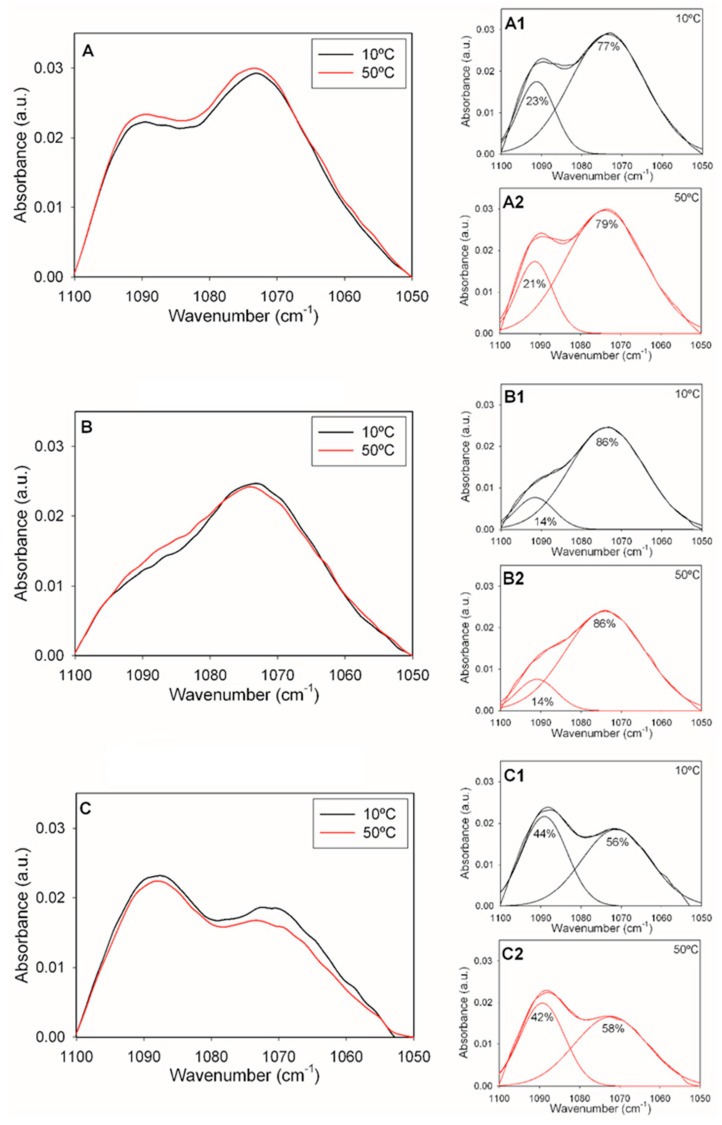
The phosphate band region (1100–1050 cm^−1^) of the IR spectrum of aqueous dispersions of (**A**) DOPE-mal, (**B**) ePC:DOPE:PI (35:55:10) or (**C**) ePC:DOPE:DOPEmal:PI (35:25:30:10). (**A1**,**A2**) DOPE-mal spectra, respectively, at 10 and 50 °C, each fitted to two components. (**B1**,**B2**) ePC:DOPE:PI spectra, respectively, at 10 and 50 °C, each fitted to two components. (**C1**,**C2**) ePC:DOPE:DOPEmal:PI spectra, respectively, at 10 and 50 °C, each fitted to two components. The percentage fractional areas are indicated for each component.

Arrondo et al. [19] studied the 1000–1300 cm^−1^ region of the IR spectrum of dipalmitoyl phosphatidylcholine (DPPC) in H_2_O and found absorption bands characteristic of the phosphate group with maximum wavenumbers at 1060, 1086 and 1222 cm^−1^. Figure 3 shows the region of the IR spectrum corresponding to the phosphate vibrations of the three samples described above, retrieved at 10 °C and 50 °C. In all cases, bands with maxima near 1070 and 1090 cm^−1^ were observed. The band equivalent to 1222 cm^−1^ in DPPC could not be detected in our case, because our samples were prepared in D_2_O, and the O-D vibration would overlap with the putative 1222 cm^−1^ component. For pure DOPE-mal (Figure 3A), the 1072 band was more intense than the one at 1092 cm^−1^, and the same was true for the ePC:DOPE:PI mixture (Figure 3B) but not for the ePC:DOPE:DOPE-mal:PI one (Figure 3C). The decomposed spectra in the right-hand panels in the figure (A1, A2, B1, B2, C1 and C2) confirmed the virtual absence of change with the temperature in this spectral region. The spectrum of ePC:DOPE:DOPE-mal:PI could not be interpreted as the addition of the other two; rather, the observations in Figure 3 would indicate a PE-mal-induced conformational change in the interfacial phosphate region of the ePC:DOPE:PI bilayers.

**Figure 4:**
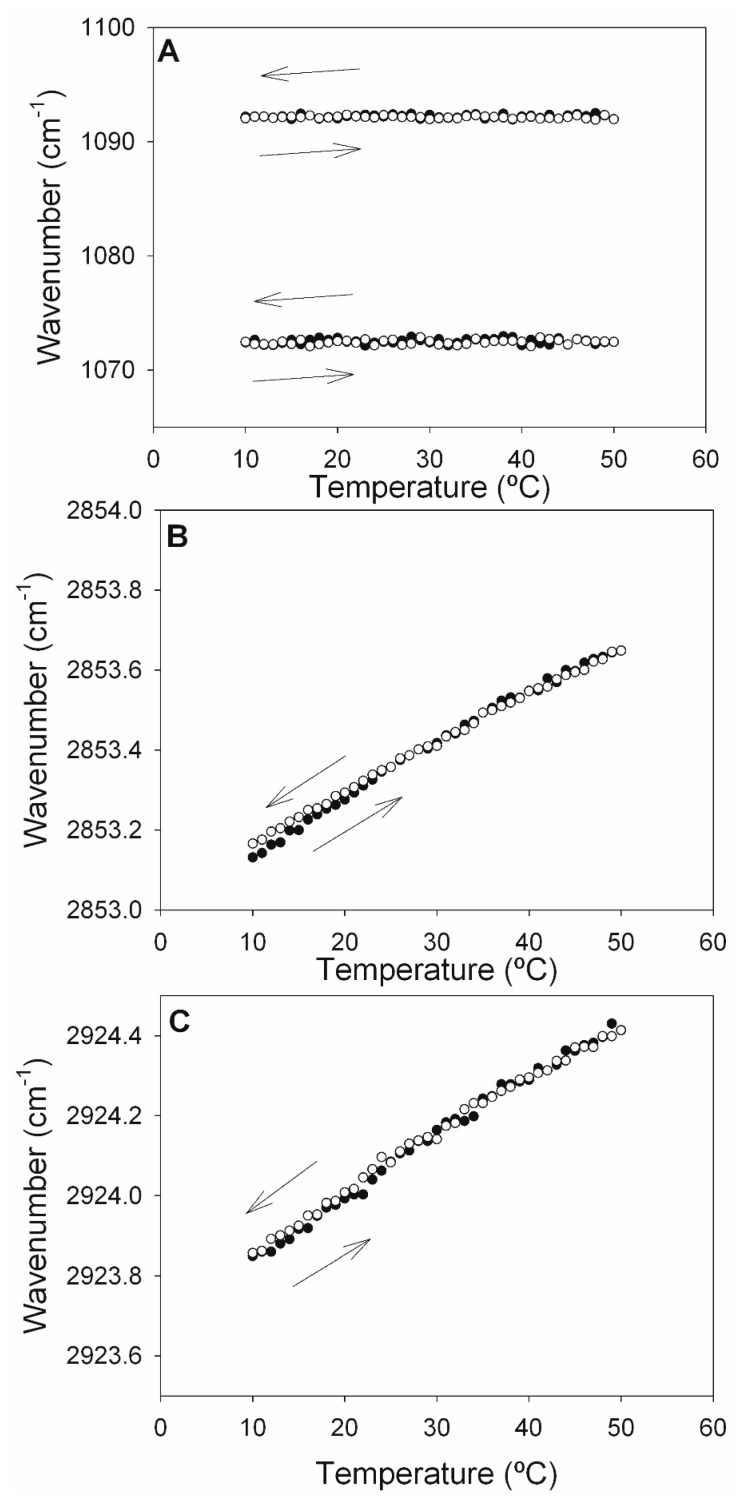
Maximum wavelengths of components of the pure DOPE-mal IR spectrum as a function of temperature. (**A**) Symmetric and asymmetric phosphate stretching vibrations. (**B**) Symmetric methylene C-H stretching vibrations. (**C**) Asymmetric methylene C-H stretching vibrations. Data from heating (full circles) and cooling (empty circles) runs are shown.

The change in the DOPE-mal band positions as a function of the temperature was explored in the 10–50 °C range, in successive heating and cooling runs (Figure 4). The data in Figure 4A confirmed the temperature independence of the 1072 and 1092 cm^−1^ phosphate bands discussed above. The cooling and heating runs were mirror images of each other, thus ensuring the reversibility (excluding hysteresis) of the process. Figure 4B,C depict the temperature-dependent shift of, respectively, the symmetric and asymmetric C-H stretching vibrations of the methylene groups in our samples. The linear shifts, toward higher wavenumbers, in the heating runs were interpreted as a gradual increase in bilayer disorder [19]. The absence of discontinuities was an indication of a lack of phase transitions under these conditions.

**Figure 5:**
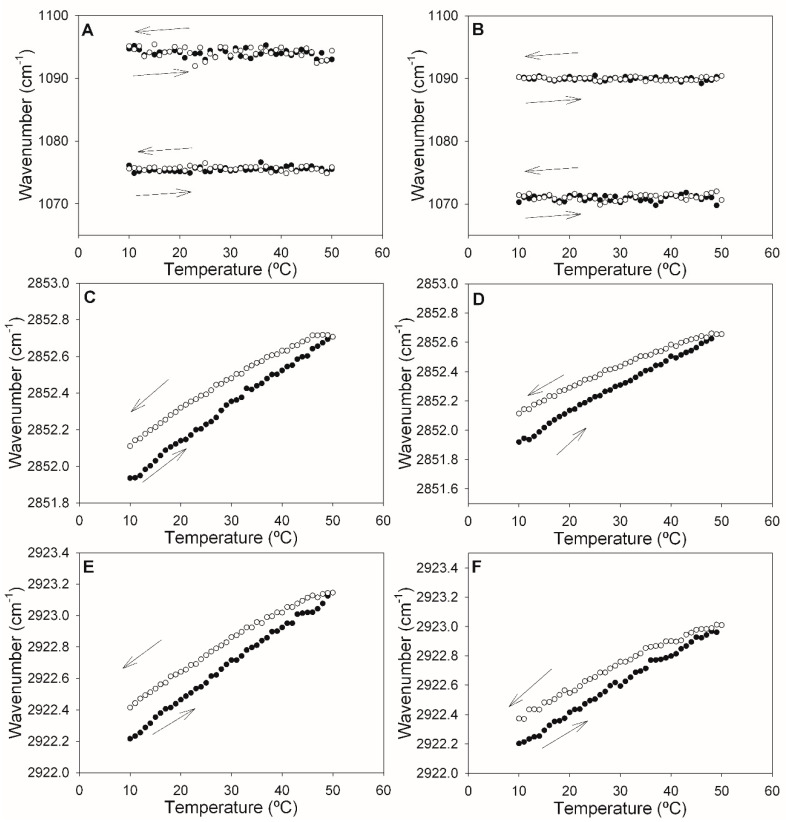
Maximum wavelengths of components of the ePC:DOPE:PI (35:55:10) IR spectrum (**A**,**C**,**E**), or of the ePC:DOPE:DOPEmal:PI (35:25:30:10) IR spectrum (**B**,**D**,**F**), as a function of temperature. (**A**,**B**) Symmetric and asymmetric phosphate stretching vibrations. (**C**,**D**) Symmetric methylene C-H stretching vibrations. (**E**,**F**) Asymmetric methylene C-H stretching vibrations. Data from heating (full circles) and cooling (empty circles) runs are shown.

The results from comparing the samples without or with DOPE-mal can be seen in Figure 5. The phosphate bands (Figure 5A,B) remain unshifted by the temperature, as was the case for the pure DOPE-mal (Figure 4A). However, in the presence of DOPE-mal (Figure 5B), both bands are shifted toward higher frequencies (lower wavenumbers), by about 5 cm^−1^, relative to the sample without DOPE-mal (Figure 5A). Phosphate band-shifts toward lower wavenumbers have been associated to increased hydration at the lipid–water interface [19].

The data corresponding to the methylene group stretching vibrations (Figure 5C–F) did not suggest any effect of DOPE-mal on the ePC:DOPE:PI bilayers. In all the cases described in Figure 5, the cooling and heating runs indicated good reversibility of the thermal effects.

### 2.3. Fluorescence Studies

Two important membrane properties that can be conveniently explored with fluorescence techniques are membrane permeability and bilayer fluidity. The putative effects of PE-mal on membrane permeability were assessed using both fluorescence spectroscopy and fluorescence microscopy. In these experiments, LUVs composed of ePC:DOPE:PI (35:55:10) were used, in which part of the DOPE was substituted with DOPE-mal when required.

**Figure 6:**
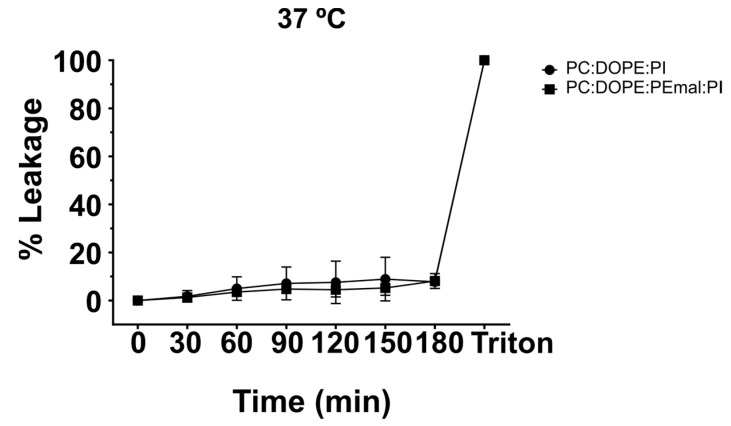
Fluorescence spectroscopy assessment of vesicle permeability. Time courses of ANTS/DPX leakage from large unilamellar vesicles (LUVs) of ePC:DOPE:PI (35:55:10) (circles) or ePC:DOPE:DOPEmal:PI (35:25:30:10) (squares), measured at different temperatures. The 100% leakage is measured after Triton X-100 addition.

For the fluorescence spectroscopy experiments, LUVs composed of either ePC:DOPE:PI (35:55:10) or of ePC:DOPE:DOPE-mal:PI (35:25:30:10) were used. They were loaded with the water-soluble probes ANTS/DPX [20]. The vesicles were transferred to a spectroscopic cuvette in an iso-osmotic buffer, and the fluorescence was recorded for 3 h. Membrane permeabilization should be detected as an increase in ANTS fluorescence due to the dissociation of the ANTS/DPX complex. In our case, the ANTS release in the first 3 h was minimal (Figure 6), and the presence of PE-mal made no difference. The 100% release was measured after addition of Triton X-100 to solubilize the bilayers. The data in Figure 6 were retrieved at 37 °C. The data recorded at 20 °C and 50 °C are shown in Supplementary Figure S1; again, the PE-mal failed to show any effect at those temperatures. The quantitative data are summarized in Table 3.

**Figure 7:**
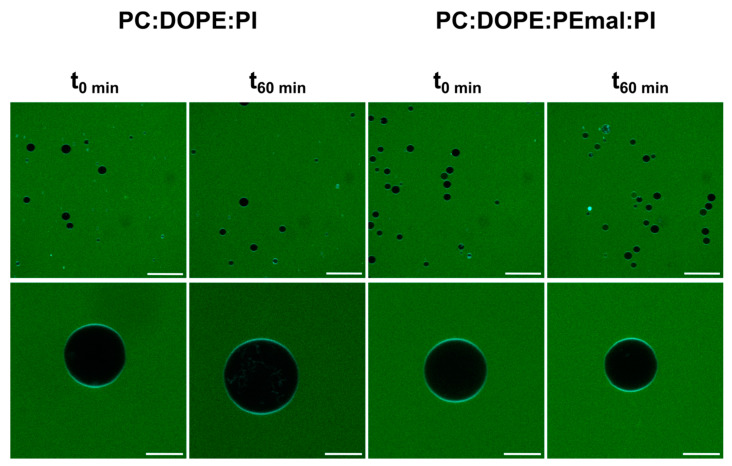
Microscopic assessment of vesicle permeability. GUVs of ePC:DOPE:PI (35:55:10) or ePC:DOPE:DOPEmal:PI (35:25:30:10) incubated with Alexa 488. Bar (broad field): 50 µm. Bar (individual GUV): 10 µm.

For fluorescence microscopy assays of the membrane permeability, GUVs of either of the above compositions were placed in the observation chamber (Figure S2), and the water-soluble dye Alexa 488 was added to the medium. Confocal microscopy images were retrieved at 0 and 60 min after the Alexa 488 addition (Figure 7). At time 0 min, the inner compartment of the GUVs appeared dark, due to the lack of the fluorescent probe inside. At time 60 min, the inner compartments remained dark, both in the presence and absence of PE-mal, indicating that the latter lipid did not facilitate the membrane permeation of Alexa 488. The results in Figure 6 and Figure 7 concur in showing that PE-mal, under our experimental conditions, does not increase membrane permeability.

**Figure 8:**
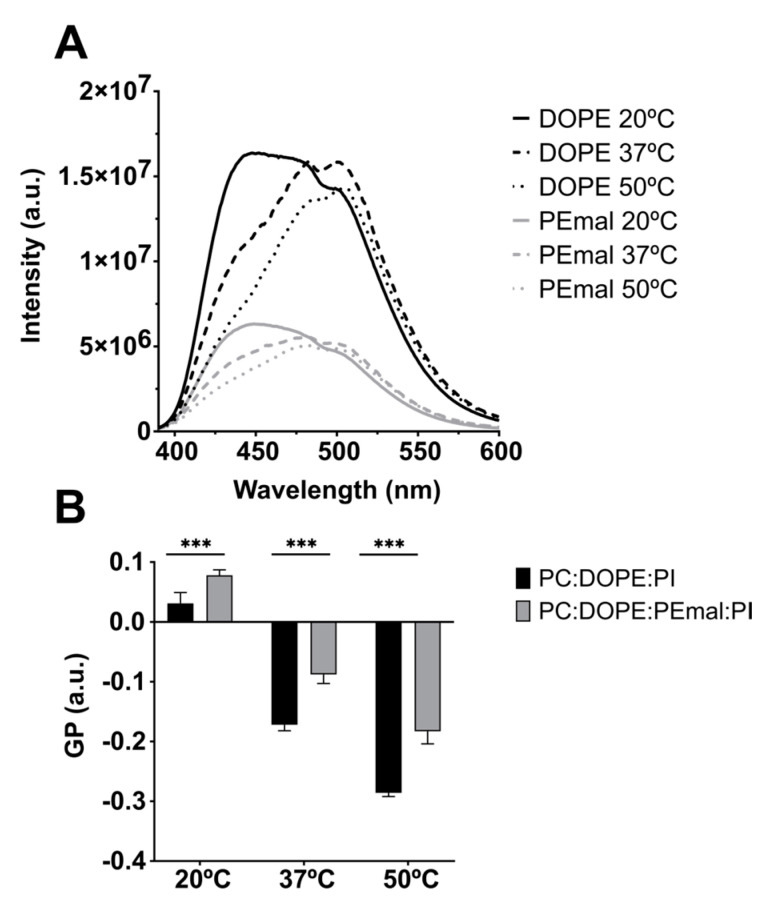
Changes in membrane fluidity caused by DOPEmal, measured using Laurdan fluorescence. Bilayer compositions: ePC:DOPE:PI (35:55:10) (black) and ePC:DOPE:DOPEmal:PI (35:25:30:10) (grey). Laurdan general polarization (GP) was measured in MLVs. (**A**) Representative emission spectra. (**B**) GP value ± S.D. (*n* = 3). Student’s significance *t*-test: *** indicates *p* < 0.001.

The PE-mal effects on membrane fluidity were explored using three different probes, namely, Laurdan, DPH and TMA-DPH. Laurdan is a solvatochromic probe that changes its emission spectra depending on the polarity of the environment, so that when the probe is in a more rigid environment, its maximum emission shifts toward the blue, and when it is present in a more fluid phase, the maximum emission shifts toward the red. The Laurdan emission spectra in the bilayers composed of either ePC:DOPE:PI (35:55:10) or of ePC:DOPE:DOPE-mal:PI (35:25:30:10), retrieved at 20 °C, 37 °C or 50 °C, are depicted in Figure 8A. Increasing the T led to a higher fluidity and thus to a higher maximum emission wavelength. The Laurdan generalized polarization (GP), computed as described in the Methods, increased significantly in the presence of PE-mal (Figure 8B), suggesting that the probe was in a more rigid environment under those conditions.

**Figure 9:**
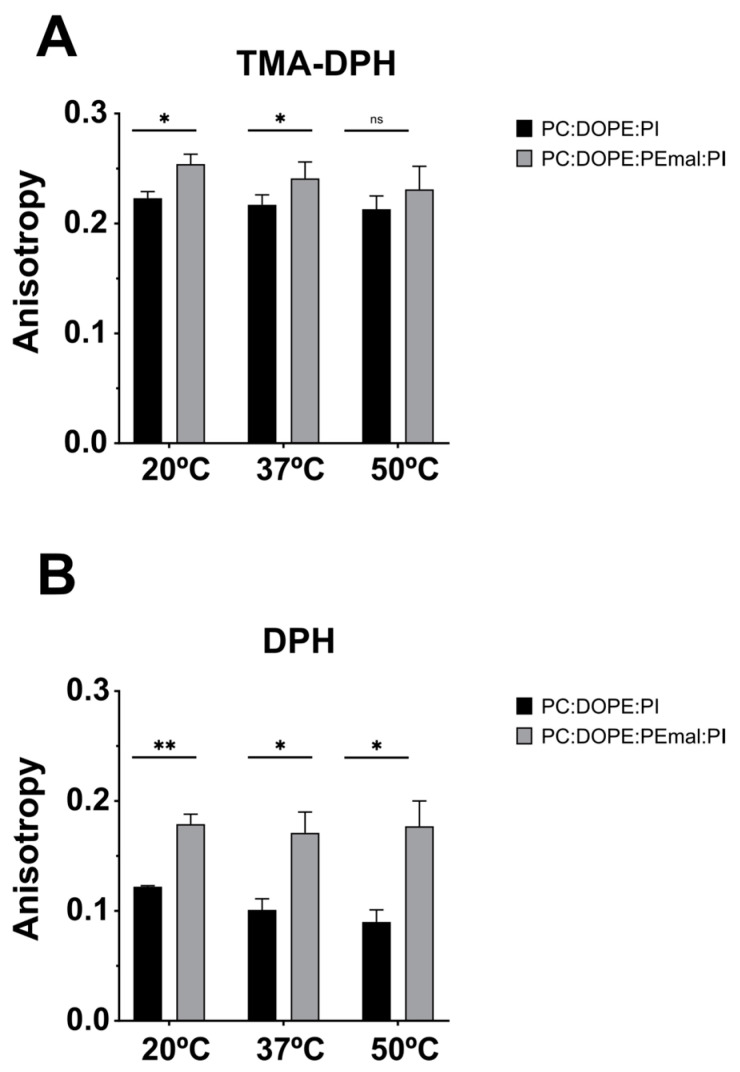
TMA-DPH and DPH anisotropy values in membranes with or without PEmal. Bilayer compositions: ePC:DOPE:PI (35:55:10) (black) and ePC:DOPE:DOPEmal:PI (35:25:30:10) (grey). Anisotropy measurements with TMA-DPH (**A**) or DPH (**B**). The 0.0375 mM LUV (20 °C and 37 °C) and 0.075 mM LUV (50 °C). Student’s significance *t*-test: ** *p* < 0.01; * *p* < 0.05; ns, nonsignificant.

TMA-DPH, when incorporated into membranes, remains anchored at the lipid–water interface, close to the lipid–water boundary, while DPH partitions freely in the bilayer hydrophobic matrix. Both probes provided consistent information when added to the ePC:DOPE:PI (35:55:10) or ePC:DOPE:DOPE-mal:PI (35:25:30:10) bilayers (LUV). In all the cases, at 20 °C, 37 °C and 50 °C, the TMA-DPH and DPH fluorescence emission anisotropies were significantly higher in the presence of PE-mal (except for TMA-DPH at 50 °C, in which case the difference was not significant) (Figure 9). Increased DPH or TMA-DPH anisotropies are indicative of an increased bilayer order [21]. In conclusion, all three fluorescent probes tested show agreement in reporting an increased rigidity (decreased fluidity) as a result of PE-mal incorporation in the lipid bilayer.

## 3. Discussion

The use of model systems is an essential tool in the understanding of biological processes at the cellular and molecular level; autophagy is a prominent example of this approach. One of the early stages of autophagy includes the expansion of the so-called phagophore, giving rise to the autophagosome (AP). An essential step in this event is the covalent binding of one member of the Atg8 family of autophagy proteins to a phospholipid, usually phosphatidyl ethanolamine (PE), in the nascent AP. In the cell, this is achieved by the concerted action of several Atg proteins and requires ATP hydrolysis. However, a model system has been developed, and extensively used, in which a chemically synthesized maleimide derivative of PE (PE-mal) is allowed to react with a Cys group of an Atg8 protein, thus achieving the protein covalent binding to a membrane lipid [2,8,13]. In spite of the results showing that the chemically lipidated Atg8 is functionally similar to the one processed via the enzyme cascade [6,8], some doubts have been raised about the exact equivalence of the two procedures, enzymatic and chemical. One basic question refers to whether or not PE-mal behaves in the lipid bilayer in the same way as PE [15]. In order to clarify the situation, a number of biophysical measurements have been performed in which bilayers containing only PE, or mixtures of PE + PE-mal, were studied using calorimetric and spectroscopic procedures. The specific vesicle compositions used were ePC:DOPE:PI (35:55:10) or ePC:DOPE:DOPE-mal:PI (35:25:30:10), largely because they have often been used in autophagy studies.

Some results support the idea that PE and PE-mal are equivalent from the point of view of the membrane structure. This is the case of the calorimetric data obtained when PE or PE-mal were mixed at 30 mol% with the bilayer-forming DPPC (Figure 1) or the IR spectral data of methylene stretching vibrations of the ePC:DOPE:PI (35:55:10) or ePC:DOPE:DOPE-mal:PI (35:25:30:10) bilayers (Figure 5C–F). Differential scanning calorimetry and methylene IR stretching vibrations often give rise to concordant results, in spite of their very diverse physical foundations, see, e.g., [22,23]. Importantly, DOPE-mal was as inactive as DOPE in facilitating vesicle membrane permeability (Figure 6, Figure 7 and Figure S1).

Nevertheless, other experiments discussed in this paper reveal differences in the properties of PE and PE-mal when incorporated into membranes. In contrast with the behavior in experiments based on DPPC, PE-mal was much more potent than PE in perturbing the thermotropic transitions of DEPE (Figure 2 and Table 2). (Note that pure DPPC generates more stable bilayers than DEPE.)

Another important difference between DOPE and DOPE-mal is that the latter increases (makes more positive) the Laurdan GP values (Figure 8). Similarly, the anisotropy values of DPH and of TMA-DPH are higher in the presence of PE-mal than in its absence (Figure 9). The Laurdan and DPH probe behavior indicates a higher rigidity of the DOPE-mal-containing membranes. Maruyama et al. [15] found that Atg8 lipidation on the membrane induced the deformation of prolate GUVs into spheres with an out-bud. However, chemical linking of mCherry–Atg8 to PE possessing a reactive maleimidomethyl-cyclohexane-carboxamide phospholipid in the prolate GUVs did not cause any morphological changes. The observations in Figure 8 and Figure 9, of an increased rigidity in bilayers containing PE-mal, could explain the observed resistance to the morphological changes in the GUVs.

In conclusion, the partial substitution of PE for PE-mal in bilayers composed of ePC:DOPE:PI induces some quantitative changes in certain (not all) physical properties of the bilayer. No qualitative changes, i.e., neither the emergence nor the total loss, of bilayer properties were perceived. With respect to the question on whether PE-mal-mediated chemical modification of Atg8 is an acceptable procedure in in vitro autophagy assays, these authors consider that it does not depart from the physiological situation more than the use of membrane models in itself. However, as in all model studies, the limitations, as described above, should not be dismissed. It would also be pertinent to note that, if PE-mal increases membrane rigidity, the observed changes in the membrane architecture (e.g., vesicle tethering, intervesicular lipid mixing and vesicle–vesicle fusion) [8] would in any case be slightly hampered by PE-mal, without the risk of observing PE-mal-induced new, artifactual effects.

## 4. Materials and Methods

### 4.1. Materials

L-α-phosphatidylcholine from hen egg yolk (ePC, 840,051), 1,2-dipalmitoyl-sn-glycero-3-phosphatidylcholine (DPPC, 850,355), liver phosphatidylinositol (PI, 840,042), 1,2-dioleoyl-*sn*-glycero-3-phosphatidylethanolamine-N-lissamine rhodamine B sulfonyl (Rho-PE, 810,150), 1,2-dioleoyl-*sn*-glycero-3-phosphatidylethanolamine (DOPE, 850,725), 1,2-dioleoyl-*sn*-glycero-3-phosphoethanolamine-N-[4-(p-maleimidomethyl) cyclohexane-carboxamide] (DOPE-mal, 780,201), 1,2-dielaidoyl-*sn*-glycero-3-phosphoethanolamine (DEPE, 850,726) and 1,2-dioleoyl-*sn*-glycero-3-phosphoethanolamine-N-(7-nitro-2-1,3-benzoxadiazol-4-yl) (NBD-PE, 810,145) were purchased from Avanti Polar Lipids, Inc. (Alabaster, AL, USA). p-Xylene-bis-pyridinium bromide (DPX, X-1525) and 8-aminonaphthalene-1,3,6-trisulfonic acid and disodium salt (ANTS, A350) were purchased from Thermo Fisher Scientific (Waltham, MA, USA). 1-[6-(Dimethylamino)naphthalen-2-yl]dodecan-1-one (Laurdan), 1,6-diphenyl-1,3,5-hexatriene (DPH) and N,N,N-trimethyl-4-(6-phenyl-1,3,5-hexatrien-1-yl)phenyl-ammonium (p-toluenesulfonate) (TMA-DPH) were supplied by Molecular Probes (Eugene, OR, USA).

### 4.2. Vesicle Preparation

The appropriate lipids were mixed in organic solution and the solvent was evaporated to dryness under an N2 stream. Then, the sample was kept under vacuum overnight to remove solvent traces. Lipid compositions are given as mole ratios. The lipids were swollen in buffer (150 mM NaCl, 50 mM Tris-HCl, pH 7.5) in order to obtain multilamellar vesicles (MLVs). Large unilamellar vesicles (LUVs) were produced from MLVs according to the extrusion method described by Mayer et al. [24]. They were subjected to 10 freeze/thaw cycles and then extruded (LIPEX Liposome Extrusion System (Transferra Nanosciences, Burnaby, BC, Canada)) using 0.1 μm pore size Nuclepore filters (Whatman, 110,605 (Little Chalfont, UK)). Vesicle size was checked by quasi-elastic light scattering using a Malvern Zeta-Sizer 4 spectrometer (Malvern Instruments, Malvern, UK). LUVs had an average diameter of ≈100 nm. Phospholipid concentration was determined by phosphate analysis [25].

Giant unilamellar vesicles (GUVs) were formed in a PRETGUV 4 chamber supplied by Industrias Técnicas ITC (Bilbao, Spain), using the modified electroformation method first developed by Angelova and Dimitrov [26]. A lipid stock of the desired GUV composition was prepared at 1 mM in chloroform: methanol (2:1, *v*/*v*). Labeling was carried out by pre-mixing the desired fluorescent probe (Rho-PE) with the lipids in organic solvent. The fluorescent probe concentration was 0.5 mol% Rho-PE. In total, 20 µL of the stock was placed on indium tin oxide (ITO)-coated glass electrodes (10 µL on each conducting surface) and kept under vacuum overnight to remove solvent traces. Then, 300 mM sucrose was added to the lipid mixture. The electroformation protocol consisted of 10 Hz, 1 Vrms for 90 min and ≈10 °C above the transition temperature of the lipid mixture. Finally, GUVs were transferred to the visualizing chamber, where 150 mM NaCl, 50 mM Tris-HCl and pH 7.5 buffer was added. Images were acquired on a Leica SP5 confocal microscope with a 63× Water Planar Apochromat 1.2 NA objective at 23 °C. The excitation and emission wavelengths used for Rho-PE were 514 nm and 580–600 nm, respectively [27].

### 4.3. Differential Scanning Calorimetry (DSC)

A nano-DSC (TA Instruments, New Castle, DE, USA) was used to perform these experiments. Multilamellar vesicles (MLVs), final concentration 4–5 mM, were prepared with the appropriate lipid compositions. Then, the sample was hydrated by adding the buffer solution in successive small amounts, helping the dispersion by stirring with a glass rod at a temperature above the transition temperature of the lipid mixture. The vesicles were homogenized by forcing the sample 50–100 times through a narrow tube (0.5 mm internal diameter, 10 cm long) between two syringes. Before loading the MLV sample into the appropriate calorimeter cell, both the lipid and buffer solutions were degassed. In total, 0.5 mL suspension containing 4–5 mM total lipid concentration was loaded into the calorimeter. For all samples, six successive heating scans at 45 °C/h, between 0 and 100 °C, were performed. The thermodynamic parameters were obtained with the software Origin 7.0 (MicroCal, Malvern, UK) provided with the nano-DSC. Using the software, the baseline was subtracted from the sample scan and a sigmoid adjustment was performed to obtain a flat line before and after the transition temperature. Finally, the phospholipid concentration was determined using a phosphorus assay and the thermogram was normalized according to the measured concentration. Gaussian fittings of the thermograms were performed to visualize the different components in each endotherm. Further information on calorimetric studies of phospholipid-containing samples can be retrieved elsewhere [28].

### 4.4. IR Spectroscopy

Infrared spectra were recorded in a Thermo Nicolet Nexus 5700 (Thermo Fisher Sccientific, Waltham, MA, USA) spectrometer equipped with a liquid nitrogen-refrigerated mercury-cadmium-telluride detector and a Peltier-based temperature controller (TempComp, BioTools Inc., Wauconda, IL, USA). A 25 μL sample aliquot was deposited on a 25 μm optical-path calcium fluoride cell (BioCell, BioTools Inc., Wauconda, IL, USA) that was sealed with a second cell. Typically, 370 scans for each, background and sample, were collected at 2 cm^−1^ resolution and averaged after each minute. Temperature was increased at a rate of 1 °C/min. Data treatment and band decomposition of the original amide I and phosphate bands were performed as described elsewhere [29,30,31].

### 4.5. Vesicle Leakage Assessment (Fluorescence Spectroscopy)

Leakage of vesicle contents was monitored by the ANTS/DPX assay [20] at pH 7.5. ANTS emission was monitored at 520 nm with the excitation wavelength set at 355 nm (slits at 2 nm). To establish the 100% leakage signal, Triton X-100 (St. Louis, MO, USA) was added to a concentration of 1%. Details for the vesicle contents leakage assay can be found in Goñi et al. [32].

### 4.6. Vesicle Permeability Assessment (Fluorescence Microscopy)

GUV permeability was assessed as follows. GUVs were placed in the visualizing chamber, with the appropriate buffer, as described above. The water-soluble fluorescent dye Alexa 488 (5 µM) (Cambridge, UK) was added to the vesicle suspension. The system was examined under a Leica TCS SP5 II fluorescence microscope (Leica Microsystems GmbH, Wetzlar, Germany), with a 63× Water Planar Apochromat 1.2 NA objective, at room temperature, for up to 60 min. Excitation and emission wavelengths were 499 and 520 nm, respectively. Dye entry into the vesicles should be an indication of vesicle membrane permeabilization.

### 4.7. Membrane Fluidity Measurements (Laurdan and DPH)

Laurdan membrane fluidity measurements were conducted on MLV membranes formed from the appropriate lipid mixtures. Laurdan fluorescence general polarization (GP) is often used as an indication of membrane fluidity/rigidity. When a lipid membrane is in the gel phase the Laurdan emission peaks at 440 nm, whereas in the liquid crystalline phase the spectrum is red-shifted to approximately 490 nm [33,34]. MLVs were mixed at a final 110:1 lipid:Laurdan molar ratio. Lipid extracts in chloroform:methanol (2:1) were mixed with Laurdan and the solvent was evaporated to dryness under a stream of N2. Then, the sample was kept under vacuum for 2 h to remove solvent traces and the lipids were swollen in buffer (150 mM NaCl, 50 mM Tris-HCl and pH 7.5). Final concentrations were 82.5 µM lipid and 0.75 µM Laurdan. Fluorescence measurements were performed using a QuantaMaster 40 spectrofluorometer (Photon Technology International, Lawrenceville, NJ, USA). Laurdan generalized polarization (GP) value was computed using the following equation:

GP = I_440_ − I_490_/I_440_ + I_490_ (1)

where I_440_ and I_490_ represent the fluorescence intensity emitted at 440 nm and 490 nm, respectively [32].

For DPH or TMA-DPH fluorescence anisotropy measurements, 8 µM DPH, or TMA-DPH, (in DMSO) was added to pre-formed LUVs. Final DMSO concentration was 11 µg/mL. Final lipid concentration was 0.0375 mM LUV (20 °C and 37 °C) and 0.075 mM LUV (50 °C). The system was left incubating for 90 min at room temperature. Fluorescence anisotropy measurements were performed using a FluoroMax-3 spectrofluorometer (Horiba Jobin Yvon, Edison, NJ, USA) with polarizers in the excitation and emission channels and equipped with a type L measurement system. The instrument software computed anisotropies for each experimental point, automatically correcting for the G factor. The FluoroMax-3 was equipped with thermally regulated holders that allowed measurements at 20 °C and 37 °C. The wavelengths of excitation and emission for DPH or TMA-DPH were 360 and 430 nm, respectively. To avoid light scattering and inner filter effects, fluorescence anisotropy was measured on increasingly diluted samples. Only when anisotropy values remained constant with further dilution were they recorded [35].

## Figures and Tables

**Table 1:** Thermodynamic parameters of the DSC gel–fluid transitions depicted in Figure 1. Five heating and cooling scans were performed for each sample; values in this table were taken from the final scan. Average values ± S.D. (*n* = 3), except for pure DPPC (*n* = 1).

Sample	T_m_ (°C)	T_1/2_ (°C)	ΔH (kcal/mol)
DPPC:DOPEmal (70:30)	31.9 ± 0.8	10.1 ± 0.2	6.8 ± 1.7
DPPC:DOPE (70:30)	34.5 ± 0.7	8.9 ± 0.4	7.2 ± 0.7
DPPC	41.9	0.7	9.0

**Table 2:** Thermodynamic parameters of the thermotropic transitions depicted in Figure 2. Five heating and cooling scans were performed for each sample; values in this table were taken from the final scan. Average values ± S.D. (*n* = 3). The letters a, b and c correspond to the individual thermal components, indicated in the figure by dashed lines, in increasing order of transition temperatures.

Sample	Transition	T_m_ (°C)	T_1/2_ (°C)	ΔH (kcal/mol)
DEPE:PE-mal (70:30)	1			
	a	15.7 ± 3.6	14.6 ± 3.7	6.6 ± 4.2
	b	25.8 ± 2.8	10.5 ± 1.9	4.7 ± 2.9
	c	33.7 ± 2.9	4.7 ± 2.9	1.4 ± 1.6
DEPE:DOPE (70:30)	1			
	a	26.1 ± 1.3	6.8 ± 2.1	1.0 ± 0.5
	b	29.2 ± 0.4	3.0 ± 0.7	0.7 ± 0.3
	2			
	a	48.5 ± 0.1	3.2 ± 0.6	0.14 ± 0.06
	b	51.6 ± 0.5	4.5 ± 0.0	0.11 ± 0.03
DEPE	1	37.6 ± 0.1	0.9 ± 0.0	10.3 ± 0.4
	2	64.8 ± 0.1	3.1 ± 0.1	0.8 ± 0.0

**Table 3:** Percent aqueous contents leakage from liposomes after 180 min. LUVs of ePC:DOPE:PI (35:55:10) or ePC:DOPE:DOPEmal:PI (35:25:30:10) with encapsulated ANTS/DPX were incubated at 20 °C, 30° and 50 °C for 180 min. Average values of ANTS/DPX percent leakage ± S.D. (*n* = 3).

% Leakage (t_180_–t_0_)	20 °C	37 °C	50 °C
ePC:DOPE:PI	7.2 ± 3.0	7.8 ± 1.8	6.3 ± 5.9
ePC:DOPE:DOPEmal:PI	6.1 ± 4.3	8.1 ± 3.1	11.4 ± 2.5

## Data Availability

The data presented in this study are available on request from the corresponding author.

## Supplementary Materials

The following supporting information can be downloaded at: https://www.mdpi.com/article/10.3390/ijms242316570/s1.
