# Supplementary material for: Effects of a N-Maleimide-derivatized Phosphatidylethanolamine on the Architecture and Properties of Lipid Bilayers
Source: Int J Mol Sci. 2023 Nov 21;24(23):16570. doi: 10.3390/ijms242316570 (PMC10706405; doi:10.3390/ijms242316570)
Supplement: Supplementary file 1 [file ijms-24-16570-s001.zip › ijms-2703425-supplementary.pdf]

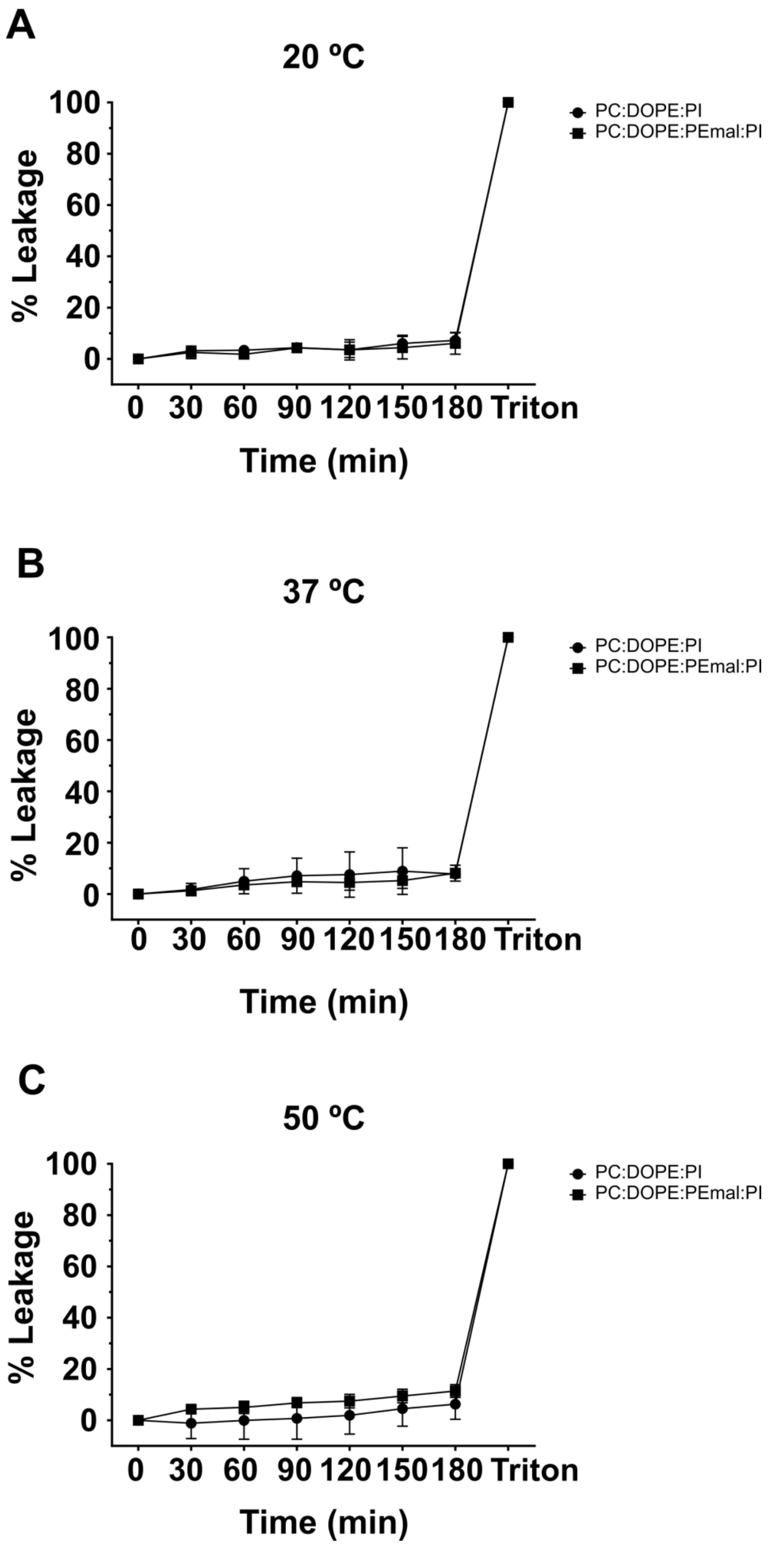

Figure S1: Fluorescence spectroscopy assessment of vesicle permeability. Time courses of ANTS/DPX leakage from large unilamellar vesicles (LUV) of ePC:DOPE:PI (35:55:10) (circles) or ePC:DOPE:PEmal:PI (35:25:30:10) (squares), measured at different temperatures.

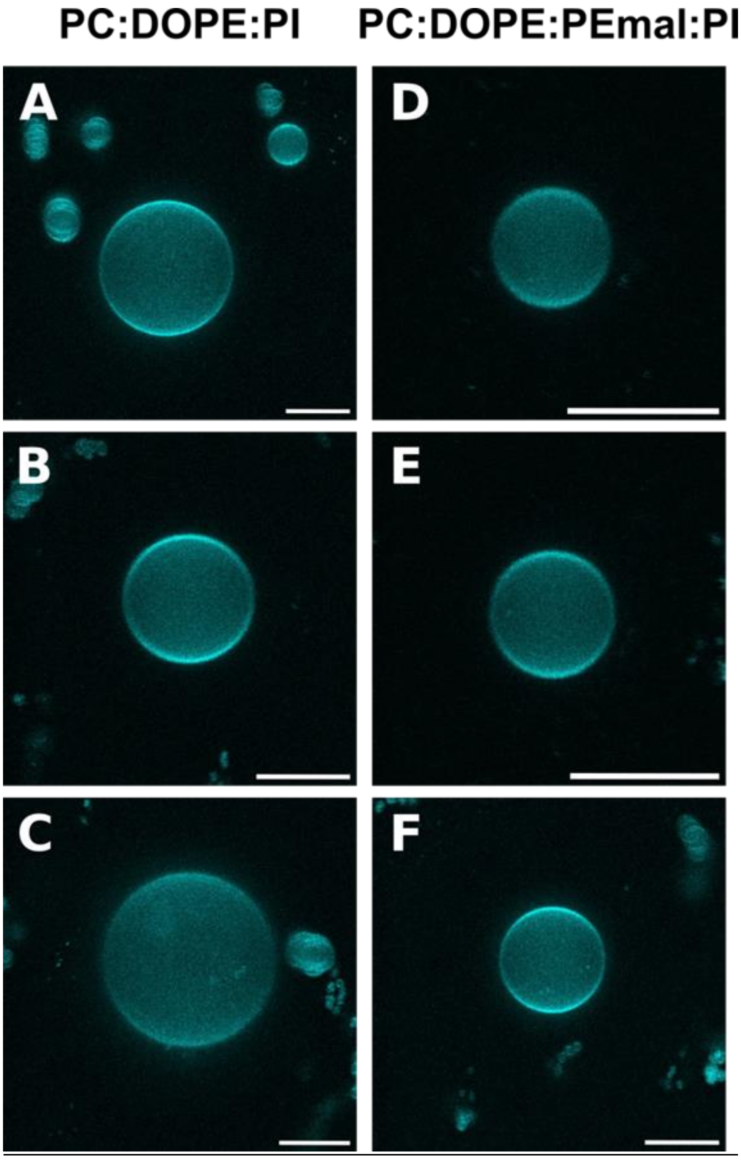

Figure S2: Representative images of giant unilamellar vesicles (GUV) composed of ePC:DOPE:PI (35:55:10) (A-C) and ePC:DOPE:PEmal:PI (35:25:30:10) (D-F) stained with Rhodamine-PE. Scale bar: 10 µm.
